# Supplementary material for: Modeling glioblastoma heterogeneity as a dynamic network of cell states
Source: Mol Syst Biol. 2021 Sep 16;17(9):e10105. doi: 10.15252/msb.202010105 (PMC8444284; doi:10.15252/msb.202010105)
Supplement: Supplementary file 5 — Source Data for Figure 3 [file MSB-17-e10105-s001.zip › Figure3A_sourcedata/GSEA_3065/hallmarks_state3.GseaPreranked.1623416274744/HALLMARK_MYOGENESIS.html]

Details for gene set HALLMARK\_MYOGENESIS[GSEA]

|  || Dataset | state3 |
| Phenotype | NoPhenotypeAvailable |
| Upregulated in class | na\_pos |
| GeneSet | HALLMARK\_MYOGENESIS |
| Enrichment Score (ES) | 0.33012843 |
| Normalized Enrichment Score (NES) | 1.2807336 |
| Nominal p-value | 0.05027933 |
| FDR q-value | 0.14565918 |
| FWER p-Value | 0.764 |
Table: GSEA Results Summary

  

Fig 1: Enrichment plot: HALLMARK\_MYOGENESIS      
 Profile of the Running ES Score & Positions of GeneSet Members on the Rank Ordered List

  

| PROBE | GENE SYMBOL | GENE\_TITLE | RANK IN GENE LIST | RANK METRIC SCORE | RUNNING ES | CORE ENRICHMENT || 1 | APP |  |  | 49 | 0.437 | 0.0363 | Yes |
| 2 | NCAM1 |  |  | 52 | 0.430 | 0.0774 | Yes |
| 3 | SCD |  |  | 108 | 0.340 | 0.1037 | Yes |
| 4 | ITGB1 |  |  | 150 | 0.310 | 0.1288 | Yes |
| 5 | COL4A2 |  |  | 232 | 0.271 | 0.1454 | Yes |
| 6 | NOTCH1 |  |  | 272 | 0.253 | 0.1652 | Yes |
| 7 | AGRN |  |  | 352 | 0.229 | 0.1781 | Yes |
| 8 | SPARC |  |  | 361 | 0.228 | 0.1991 | Yes |
| 9 | SPTAN1 |  |  | 376 | 0.223 | 0.2190 | Yes |
| 10 | ADAM12 |  |  | 455 | 0.208 | 0.2300 | Yes |
| 11 | NAV2 |  |  | 486 | 0.201 | 0.2458 | Yes |
| 12 | ATP6AP1 |  |  | 487 | 0.201 | 0.2651 | Yes |
| 13 | FDPS |  |  | 518 | 0.196 | 0.2805 | Yes |
| 14 | ITGA7 |  |  | 595 | 0.187 | 0.2897 | Yes |
| 15 | PRNP |  |  | 609 | 0.185 | 0.3059 | Yes |
| 16 | MYH9 |  |  | 688 | 0.173 | 0.3136 | Yes |
| 17 | PLXNB2 |  |  | 742 | 0.165 | 0.3233 | Yes |
| 18 | ERBB3 |  |  | 899 | 0.149 | 0.3196 | Yes |
| 19 | CLU |  |  | 988 | 0.139 | 0.3228 | Yes |
| 20 | APOD |  |  | 1113 | 0.129 | 0.3208 | Yes |
| 21 | ITGB5 |  |  | 1139 | 0.127 | 0.3301 | Yes |
| 22 | TSC2 |  |  | 1449 | 0.106 | 0.3046 | No |
| 23 | EPHB3 |  |  | 1741 | 0.089 | 0.2795 | No |
| 24 | AGL |  |  | 1793 | 0.087 | 0.2820 | No |
| 25 | GAA |  |  | 1823 | 0.085 | 0.2869 | No |
| 26 | SOD3 |  |  | 1912 | 0.080 | 0.2844 | No |
| 27 | IGFBP3 |  |  | 2112 | 0.071 | 0.2683 | No |
| 28 | AEBP1 |  |  | 2257 | 0.066 | 0.2580 | No |
| 29 | IFRD1 |  |  | 2444 | 0.059 | 0.2421 | No |
| 30 | MEF2C |  |  | 2482 | 0.058 | 0.2434 | No |
| 31 | AKT2 |  |  | 2548 | 0.055 | 0.2412 | No |
| 32 | COL6A2 |  |  | 2565 | 0.055 | 0.2446 | No |
| 33 | SH2B1 |  |  | 2656 | 0.051 | 0.2391 | No |
| 34 | LPIN1 |  |  | 2791 | 0.047 | 0.2282 | No |
| 35 | REEP1 |  |  | 2960 | 0.042 | 0.2129 | No |
| 36 | PSEN2 |  |  | 3299 | 0.033 | 0.1770 | No |
| 37 | STC2 |  |  | 3529 | 0.027 | 0.1532 | No |
| 38 | PFKM |  |  | 3564 | 0.026 | 0.1518 | No |
| 39 | SYNGR2 |  |  | 3636 | 0.025 | 0.1460 | No |
| 40 | ADCY9 |  |  | 4036 | 0.014 | 0.1013 | No |
| 41 | GSN |  |  | 4064 | 0.013 | 0.0994 | No |
| 42 | MAPK12 |  |  | 4082 | 0.013 | 0.0987 | No |
| 43 | SCHIP1 |  |  | 4109 | 0.012 | 0.0969 | No |
| 44 | TGFB1 |  |  | 4110 | 0.012 | 0.0981 | No |
| 45 | DTNA |  |  | 4203 | 0.009 | 0.0883 | No |
| 46 | SSPN |  |  | 4525 | 0.001 | 0.0513 | No |
| 47 | DMPK |  |  | 4637 | -0.002 | 0.0387 | No |
| 48 | MEF2A |  |  | 4745 | -0.005 | 0.0268 | No |
| 49 | SPEG |  |  | 4846 | -0.007 | 0.0159 | No |
| 50 | SIRT2 |  |  | 4877 | -0.008 | 0.0132 | No |
| 51 | KCNH2 |  |  | 4963 | -0.010 | 0.0044 | No |
| 52 | KIFC3 |  |  | 5146 | -0.014 | -0.0153 | No |
| 53 | DMD |  |  | 5320 | -0.019 | -0.0334 | No |
| 54 | PDE4DIP |  |  | 5321 | -0.019 | -0.0316 | No |
| 55 | BHLHE40 |  |  | 5331 | -0.019 | -0.0308 | No |
| 56 | MYO1C |  |  | 5425 | -0.022 | -0.0395 | No |
| 57 | RIT1 |  |  | 5453 | -0.023 | -0.0404 | No |
| 58 | EFS |  |  | 5486 | -0.024 | -0.0418 | No |
| 59 | HDAC5 |  |  | 5513 | -0.025 | -0.0425 | No |
| 60 | IGFBP7 |  |  | 5667 | -0.029 | -0.0574 | No |
| 61 | SLC6A8 |  |  | 5778 | -0.032 | -0.0670 | No |
| 62 | FLII |  |  | 5814 | -0.033 | -0.0678 | No |
| 63 | TPM2 |  |  | 5823 | -0.034 | -0.0655 | No |
| 64 | SORBS1 |  |  | 6085 | -0.042 | -0.0916 | No |
| 65 | MAPRE3 |  |  | 6086 | -0.042 | -0.0876 | No |
| 66 | SVIL |  |  | 6089 | -0.042 | -0.0837 | No |
| 67 | ACSL1 |  |  | 6134 | -0.044 | -0.0846 | No |
| 68 | TEAD4 |  |  | 6135 | -0.044 | -0.0804 | No |
| 69 | PKIA |  |  | 6136 | -0.044 | -0.0761 | No |
| 70 | CHRNB1 |  |  | 6146 | -0.044 | -0.0729 | No |
| 71 | MYLK |  |  | 6330 | -0.050 | -0.0892 | No |
| 72 | EIF4A2 |  |  | 6481 | -0.056 | -0.1012 | No |
| 73 | GADD45B |  |  | 6619 | -0.062 | -0.1110 | No |
| 74 | FGF2 |  |  | 6685 | -0.065 | -0.1123 | No |
| 75 | TAGLN |  |  | 6722 | -0.067 | -0.1100 | No |
| 76 | ITGB4 |  |  | 6730 | -0.067 | -0.1044 | No |
| 77 | VIPR1 |  |  | 6987 | -0.080 | -0.1263 | No |
| 78 | MRAS |  |  | 7086 | -0.086 | -0.1293 | No |
| 79 | WWTR1 |  |  | 7187 | -0.093 | -0.1319 | No |
| 80 | BAG1 |  |  | 7323 | -0.102 | -0.1377 | No |
| 81 | FKBP1B |  |  | 7352 | -0.104 | -0.1310 | No |
| 82 | SORBS3 |  |  | 7408 | -0.109 | -0.1269 | No |
| 83 | OCEL1 |  |  | 7485 | -0.116 | -0.1245 | No |
| 84 | BIN1 |  |  | 7531 | -0.120 | -0.1182 | No |
| 85 | PDLIM7 |  |  | 7630 | -0.131 | -0.1169 | No |
| 86 | CRAT |  |  | 7655 | -0.133 | -0.1069 | No |
| 87 | CKB |  |  | 7659 | -0.134 | -0.0943 | No |
| 88 | TPM3 |  |  | 7943 | -0.175 | -0.1102 | No |
| 89 | FHL1 |  |  | 8139 | -0.214 | -0.1122 | No |
| 90 | CRYAB |  |  | 8223 | -0.234 | -0.0993 | No |
| 91 | CNN3 |  |  | 8228 | -0.234 | -0.0772 | No |
| 92 | NQO1 |  |  | 8263 | -0.244 | -0.0577 | No |
| 93 | GABARAPL2 |  |  | 8419 | -0.301 | -0.0467 | No |
| 94 | MYL6B |  |  | 8618 | -0.422 | -0.0290 | No |
| 95 | CDKN1A |  |  | 8657 | -0.461 | 0.0109 | No |
Table: GSEA details [plain text format]

  

Fig 2: HALLMARK\_MYOGENESIS: Random ES distribution      
 Gene set null distribution of ES for **HALLMARK\_MYOGENESIS**
